# Molecular Understanding of Fouling Induction and Removal: Effect of the Interface Temperature on Milk Deposits

**DOI:** 10.1021/acsami.1c09553

**Published:** 2021-07-26

**Authors:** Alejandro Avila-Sierra, Holly A. Huellemeier, Zhenyu J. Zhang, Dennis R. Heldman, Peter J. Fryer

**Affiliations:** † School of Chemical Engineering, University of Birmingham, Birmingham B15 2TT, United Kingdom; ‡ Department of Food, Agricultural, and Biological Engineering, The Ohio State University, Columbus 43210 Ohio, United States; § Department of Food Science and Technology, The Ohio State University, Columbus 43210 Ohio, United States

**Keywords:** milk fouling, elevated temperature, induction, QCM-D, nanomechanical properties

## Abstract

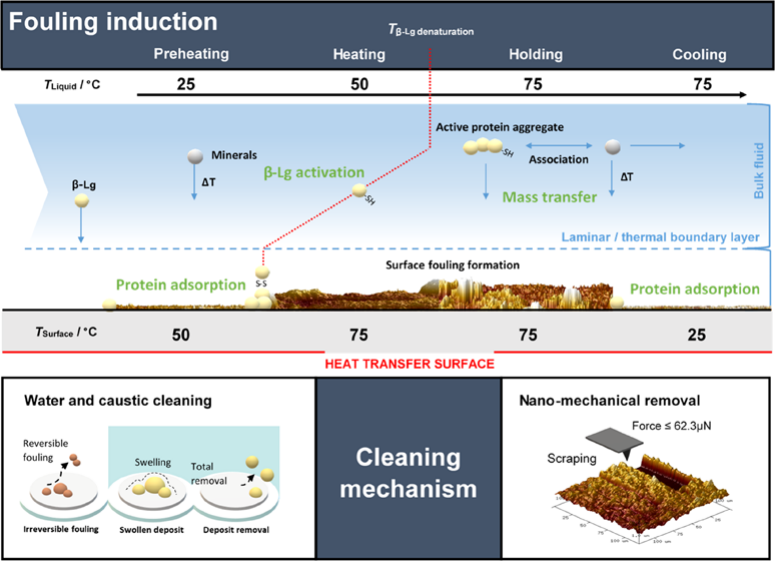

Molecular details concerning the induction phase of milk fouling on stainless steel at an elevated temperature range were established to better understand the effect of temperature on surface fouling during pasteurization. The liquid–solid interface that replicates an industrial heat exchanger (≤75°C), including four stages (preheating, heating, holding, and cooling), was investigated using both a quartz crystal microbalance (QCM-D) and a customized flow cell. We found that the milk fouling induction process is rate-limited by the synergistic effects of bulk reactions, mass transfer, and surface reactions, all of which are controlled by both liquid and surface temperatures. Surface milk foulant becomes more rigid and compact as it builds up. The presence of protein aggregates in the bulk fluid leads to a fast formation of surface deposit with a reduced Young’s modulus. Foulant adhesion and cohesion strength was enhanced as both interfacial temperature and processing time increased, while removal force increased with an increasing deposit thickness. During cleaning, caustic swelling and removal showed semilinear correlations with surface temperature (*T*_S_), where higher *T*_S_ reduced swelling and enhanced removal. Our findings evidence that adsorption kinetics, characteristics of the foulant, and the subsequent removal mechanism are greatly dependent on the temperature profile, of which the surface temperature is the most critical one.

## 1 Introduction

Deposition of proteinaceous compounds onto solid substrates is a serious concern for food processing as well other sectors such as biomedical devices and marine industry, whereby surface-anchored proteins could build-up to form thick foulant and promote biofilm growth. Pasteurization of raw milk (e.g., 71.7°C for at least 15 s) is essential to the dairy industry as it deactivates pathogens and microorganisms to ensure food safety and extend shelf life for dairy products. However, such mild heat treatment favors fouling on food-contact surfaces (e.g., stainless steel), which is a significant challenge for the food industry. Milk fouling is determined by a range of parameters: (i) properties of the milk, e.g. protein conformation/concentration, calcium concentration, pH, ionic strength; (ii) fluid dynamics such as flow rate and heat exchanger geometry, (iii) characteristics of the contact surface, for example, surface free energy, composition, and finish, and (iv) process conditions, including temperature profiles of the fluid and surface.^1^

A unique feature of fouling involved in the pasteurization process, as suggested by the previous studies, is that process temperature defines the chemical composition and extent of milk fouling:^2^ surface foulant is a soft deposit, induced by the denaturation of whey proteins (50–60%), most of which is β-lactoglobulin (β-Lg) when *T* < 100°C, but a hard composite consisting of minerals when *T* > 100°C.^3^ Upon being heated to 40°C, the native β-Lg dimer (2–5 nm^4^) starts to dissociate into monomers. With an increased temperature (40–60°C), β-Lg adjusts its tertiary structure and exposes a fraction of −SH groups, with a weak preference for aggregation. At a mildly elevated temperature (60–70°C), there is an alteration to the tertiary structure of β-Lg by breaking the non-covalent bonds, exposing the hidden S–S bond, which favors interactions between −SH groups and solid surfaces,^5−7^ leading to a notable protein aggregation and surface deposition. Although the characteristics of the solid substrate play a critical role in this process, the effect of substrate temperature on the fouling process is unclear, with little knowledge of the underpinning kinetics.

It is commonly accepted^3,8^ that the pasteurization process involves: (i) denaturation/aggregation of proteins in the bulk fluid, (ii) migration of aggregates to the surface, (iii) incorporation of proteins into the foulant layer by surface reactions, and (iv) possible re-entrainment or removal. In addition, proteinaceous fouling is often accompanied by the migration of minerals to the solid surface,^9^ which facilitates aggregation^10,11^ and enhances cohesion of the foulant.^12−14^ Jimenez and colleagues^15^ exposed stainless steel to whey protein solution and found that the metal surface was covered by homogeneous small proteinaceous clusters (60 nm) without calcium between 1 min and 2 h of processing, but aggregates of 150–350 nm building on the initial protein layer with the presence of calcium (120 ppm). The characteristics of the initial surface foulant or induction layer govern subsequent macroscopic fouling, shifting from surface-deposit to deposit–deposit interactions. It emphasizes the need to understand the mechanism and deposit properties during the induction stage of milk fouling, on which the effect of surface characteristics, in particularly the temperature, is the most critical one to investigate.

At macroscale, there have been several studies of milk fouling at elevated temperatures, in which fouling was reported to begin at wall temperatures of 60–65°C, and increase with a rising wall temperature.^1,7,12,16,17^ Blanpain-Avet and colleagues found the maximum fouling mass at bulk fluid temperatures between 71.8 and 75.5°C, suggesting that the extent of protein unfolding is not sufficient to favor irreversible aggregation amid the unfolding-limited region (<80°C), resulting in the surface deposition of unfolded protein.^16^ All of these studies confirm the influence of the temperature profile on milk fouling and highlight the role of bulk-wall temperature differences.^1,16^ Gravimetric approach or monitoring changes of the overall heat transfer coefficient was used in these studies to quantify the fouling kinetics, which is indirect and lacks molecular details. Although quartz crystal microbalance with dissipation (QCM-D) is an excellent technique widely used to measure interfacial adsorption kinetics *in situ* and to quantify viscoelastic properties of the surface-adhering layer, most QCM studies so far are limited to ambient temperature due to instrument limitations, using model protein solutions to replicate raw milk (e.g., adsorption of β-lg,^18,19^ whey protein,^20^ skimmed milk powder,^21^ and caseins^22^). At high temperatures (≤65°C), Yang and co-workers demonstrated the capability of this technique to monitor whey protein fouling and its dependence on calcium content.^14^

The present work aims to develop a molecular understanding of milk fouling during different stages of pasteurization, focusing on the effect of temperatures on the adsorption kinetics, molecular structure, mechanical properties of the milk foulants, and subsequent removal. Building upon the results generated *in situ* on 316 L stainless steel using both (i) QCM-D and (ii) a customized flow cell, a comprehensive molecular mechanism is proposed to illustrate the milk fouling induction at the liquid–solid interface of an industrial heat exchanger (25–75°C).

## 2 Materials and Methods

### 2.1 Materials

For QCM-D experiments, raw milk, provided by Waterman Dairy Facility (The Ohio State University, OH), was skimmed by centrifugation (10 000 r.p.m. and 4 °C) for 10 min, from which the liquid phase was separated and stored in a freezer (−80°C) for further use.

For flow cell experiments, a commercial whey protein concentrate (WPC) (CARBELAC 35, Carbery, Ireland) was used to prepare the model solution (10 wt %/wt; pH 6.30; 300 mL) by mixing with distilled water at room temperature for an hour.

QCM-D sensors coated with a layer of 316 stainless steel (QSX304, Nanoscience Instrument, Phoenix, AZ; fundamental frequency 4.95 MHz ± 50 kHz) were used to replicate an industrial food-contact surface. All sensors were cleaned thoroughly prior to each experiment, following the protocol suggested (Protocol C-1 of QCM user guide, Biolin Scientific).

Coupons made of 316L stainless steel (2.54 × 2.54 cm^2^) were polished to mirror finish^23^ (*R*_a_ 0.03 ± 0.01 μm; determined by white light interferometry (MicroXAM2, KLA Tencor, California)). They were cleaned using a 2.0% (wt/wt) NaOH aqueous solution at 80°C under stirring for an hour and cooled down to room temperature using a water bath. Subsequently, they were rinsed with a 1.0% (vol/vol) HCl solution, soaked in hexane (5 min) and acetone (5 min) before being dried by an air stream.^24^ All solvents used were of HPLC grade.

### 2.2 QCM-D

For QCM (Q-Sense Explorer, Nanoscience Instruments, Phoenix, AZ) experiments, skim milk samples (Section 2.1) were thawed in a water bath at room temperature, heated to a target temperature using a heating plate, and held for 10 min at the target temperature before being pumped at a flow rate of 100 μL min^–1^ over the stainless steel-coated QCM-D sensor. It is worth noting that there could be some variation with the temperature of the liquid once it enters the titanium flow module that could adjust the liquid temperature before making contact with the measurement sensor. The fouling phase lasted 15 min, followed by a deionized water rinse (10 min) to replicate the prerinse step of clean-in-place (CIP). The prerinse was followed by a chlorinated-caustic cleaning solution (0.5 wt %/wt Ecolab Principal, MN) up to total cleaning of the stainless steel sensor. The electrical conductivity and pH of the CIP chlorinated-caustic were 3.17 mS cm^–1^ and 11.5, respectively. Finally, the sensor was rinsed with deionized water to ensure total cleaning. The maximum mass and dissipation sensitivities of the QCM-D are 0.5 ng cm^–2^ and 0.04 × 10^–6^, respectively. The temperature of the sensor surface was controlled Peltier element in the chamber (QCP 101) surrounding the titanium QCM-D flow module (QFM 401, Nanoscience Instruments) as specified in Table 1. The maximum temperature recommended for this QCM-D chamber is 65°C. Each stage of interest (preheating, heating, holding, and cooling) was repeated at least twice.

**Table 1:** Temperature Profiles Implemented in the Present Work

condition	device	*T*_liquid_ (°C)	*T*_surface_ (°C)
control	QCM-D	25	25
preheating	flow cell/QCM-D	25	50
heating	flow cell	50	*T*_initial_ 75/*T*_experiment_ 62–68
	QCM-D	50	65
holding	flow cell	75	75
	QCM-D	75	65
cooling	flow cell/QCM-D	75	25

#### 2.2.1 QCM-D Data Analysis

Frequency and dissipation data was processed using the Sauerbrey model^25^ that defines frequency shift (−Δ*f*) as being directly proportional to the adsorbed mass per unit of surface area Δ*m* = (*C* · Δ*f*)/*n*, where *f* is the resonant frequency factor, *C* is a constant dependent on the piezoelectric crystal (here 0.177 mg Hz^–1^m^–2^), and *n* is the overtone number. The Sauerbrey model assumes that the surface-adsorbed layer is thin, rigid (), and evenly distributed,^26^ where *D* is the dissipation factor.

Surface adsorption/desorption kinetics were quantified for three distinct phases: fouling, caustic swelling, and caustic decay. The corresponding adsorption/desorption rates (Hz s^–1^) were extracted from the slope (Δ*f* vs time) as detailed in the SI, analyzed using linear least-squares algorithm in MATLAB (MathWorks, MA), and normalized by the Δ*f* value prior to the corresponding stage. Significant differences between rates were determined by non-overlapping 95% confidence intervals. A detailed explanation of the fitting regions is included in the SI. Removal of surface foulant was modeled as a first-order reaction: *f* = *f*_0_e^–*kt*^, where *f* is frequency in Hz, *t* is time (s), *f*_0_ is a constant (Hz), and *k* is the decay rate constant (1/s). The data was linearized (ln(*f*) vs time) to enable the use of linear least-squares fitting.

The properties of a surface-bound film can be evaluated by (i) the hydrodynamic bounding ratio (solvation), which is defined as deposit solvation ratio regarding its initial mass (Δ*f*_swelling_/Δ*f*_water rinse_), and (ii) the film viscoelasticity, defined as the ratio energy dissipation per mass (Δ*D*_swelling_/Δ*f*_water rinse_).^27^

### 2.3 Microscopic Flow Cell

The microscopic fouling setup consisted of a flow cell (details available in the SI), an integrated heating stage, and a peristaltic pump that supplies a flow rate of 6.5 mL min^–1^. The temperature of the WPC solution was controlled by an external heating plate. For cooling experiments, the flow cell was immersed in a water bath at room temperature (25°C). Surface temperature was monitored throughout the fouling cycle (up to 15 min). Coupons were taken from the flow cell in intervals of 2.5 min for a total of 15 min. The fouled samples were rinsed by 10 mL of deionized water to remove any physisorbed foulant prior to further characterization.

### 2.4 3D Laser Microscopy

Milk fouling on QCM-D sensors was characterized by a 3D laser microscope (VK-X200, KEYENCE, Itasca) at the end of the water rinse. Laser scan height was defined manually, and a final multilayer composition was carried out at 20× magnification.

### 2.5 Atomic Force Microscopy (AFM)

WPC fouling on flow cell coupons was characterized using an atomic force microscope (AFM) (Dimension 3100, Veeco, Cambridge, U.K.). Cantilevers (HQ:NSC15/AlBS AFM tip; ApexProbes, U.K.) with a high spring constant (40 N m^–1^) were employed to image the samples in contact mode under ambient conditions. Minimal setpoint voltage was maintained during the imaging process to minimize any potential disruption to the foulant formed.

An AFM-based scratching method was used to quantify the interfacial strength between the inductive foulant layer and the underlying surface. The cantilever (HQ:NSC15/AlBS AFM tip; ApexProbes, U.K.) with a conical tip (cone angle 40° and radius 8 nm) was positioned above the foulant, with a scanning angle of 90°. By controlling the applied contact pressure, removal forces were varied between 6.2 and 62.3 μN. Fouling thickness (depth of the area removed) was quantified subsequently based on the surface topography images using a Nanoscope Analysis 1.5 software (Bruker Corporation, Massachusetts).

AFM-based force spectroscopy was used to quantify the nanomechanical properties and surface adhesion of the foulant. To determine the Young’s modulus, force curves were modeled using an extension of Sneddon’s law for conical probes provided by Nanoscope analysis 1.5 (Bruker Corporation, Massachusetts), where Poisson’s ratio was assumed to be 0.477 for milk fouling^28^ and 0.270 for SS316L surface.^29^ Cantilevers with a sharp tip (HQ:NSC15/Al BS cantilever; spring constant 40 N m^–1^) were selected to eliminate the effect of roughness on surface adhesion. During the force measurements, loading force and cantilever velocity were kept at 500 nN and 2 μm s^–1^, respectively. A total of 100 contact points (10 columns × 10 rows) were surveyed at steps of 10 nm from at least three different positions per sample.

### 2.6 Micromanipulation Measurements

A micromanipulation rig^30^ was used to measure the force required to disrupt a layer of the foulant formed in the flow cell (Section 2.3) under “Cooling” conditions (Table 1) for 1 h. Traveling at 1 mm s^–1^, a force transducer (Sauter GmbH, FH5) with resolution ±1 mN scraped the foulant 1 mm above the metal surface at room temperature. Tests were repeated three times. The work per area (*W*_b_), used to quantify the cohesive properties of the deposit, is defined as *W*_b_ = 1/*A*∫_*t*0_^*t*1^*F*(*t*) · *dx*, where *F*(*t*) is the measured force, *A* is the deposit contact area, with *t*_0_ and *t*_1_ are the start and end times of the experiment.^31^

## 3 Results and Discussion

### 3.1 Fouling and Cleaning of Milk Deposits Formed under Pasteurization Conditions

The adsorption, swelling, and desorption characteristics of raw skim milk on a 316L stainless steel surface were measured *in situ* as a function of temperature profiles using QCM-D under controlled conditions as specified in Table 1.

#### 3.1.1 Milk Adsorption and Fouling Formation

**Figure 1:**
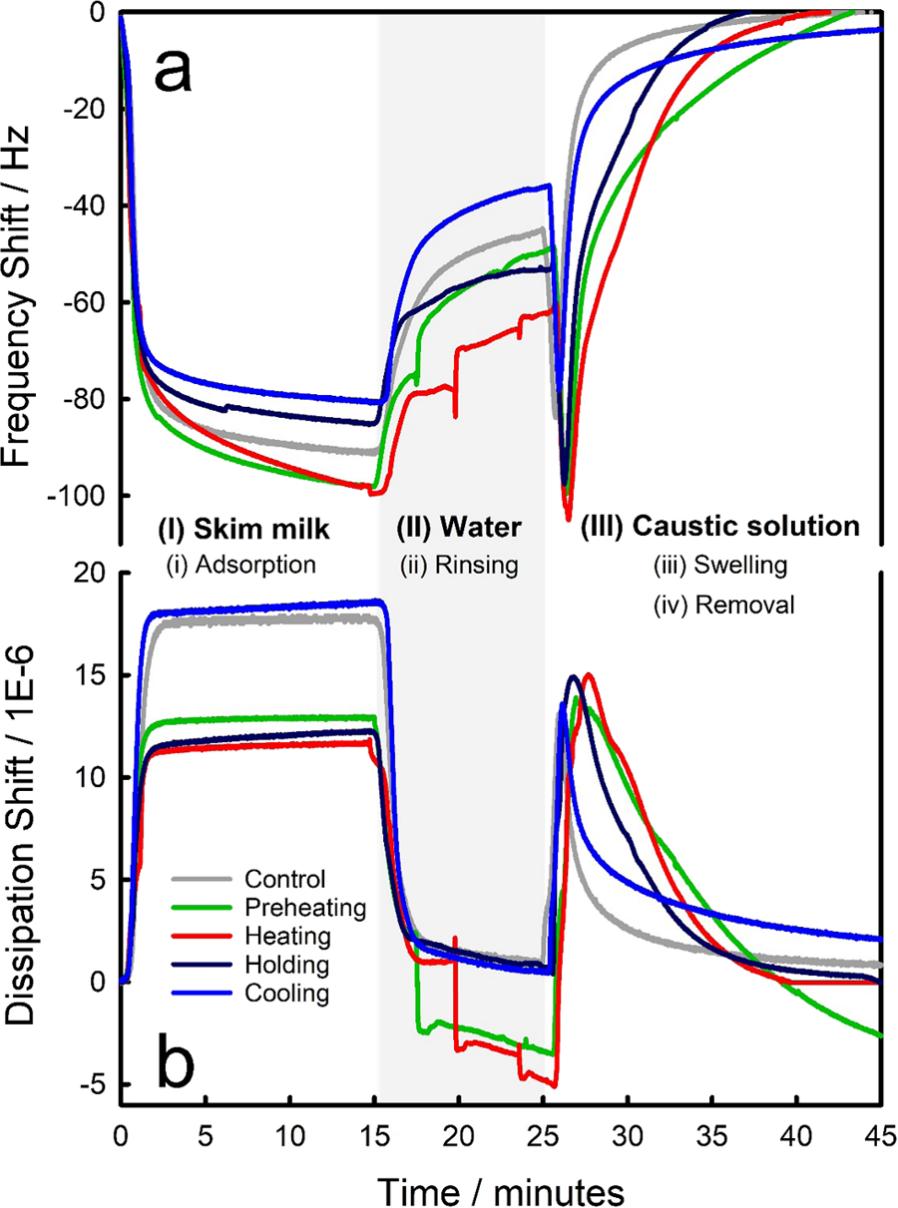
Representative fouling and cleaning cycles of raw skim milk on stainless steel surface, monitored by QCM-D as a function of temperature. Data show the averaged (a) frequency and (b) dissipation values of overtones *n* = 7, 9, and 11 under different conditions: preheating, heating, holding, and cooling, of which temperature profiles are defined in Table 1. The physical phenomena studied are: (i) adsorption of skim milk onto a stainless steel sensor (0–15 min); (ii) removal of physisorbed foulant with a water rinse (15–25 min); introduction of a chlorinated-caustic solution which causes (iii) swelling and subsequent (iv) removal of the milk fouling (25–45 min). The final phase was performed up to the total cleaning (Δ*f* ≈ 0) of the sensor.

Once the SS316 surface was exposed to raw skim milk, two stages of adsorption were observed in the first 15 min for all measurements (Figure 1a)

(i) a rapid adsorption process (0–2 min) that corresponds to the initial contact between the milk and the stainless steel surface. More than 70% of the total adsorption occurs within the first 2 min of the pasteurization process—such primary adsorption is likely limited by the diffusion kinetics of proteins through the boundary layer rather than the surface reaction itself,^32^ and
(ii) a slow process (2–15 min) that is attributed to the subsequent development of the milk foulant, which is a dynamic process that involves adsorption/desorption of milk proteins and reconfiguration of their interfacial conformation.

**Figure 2:**
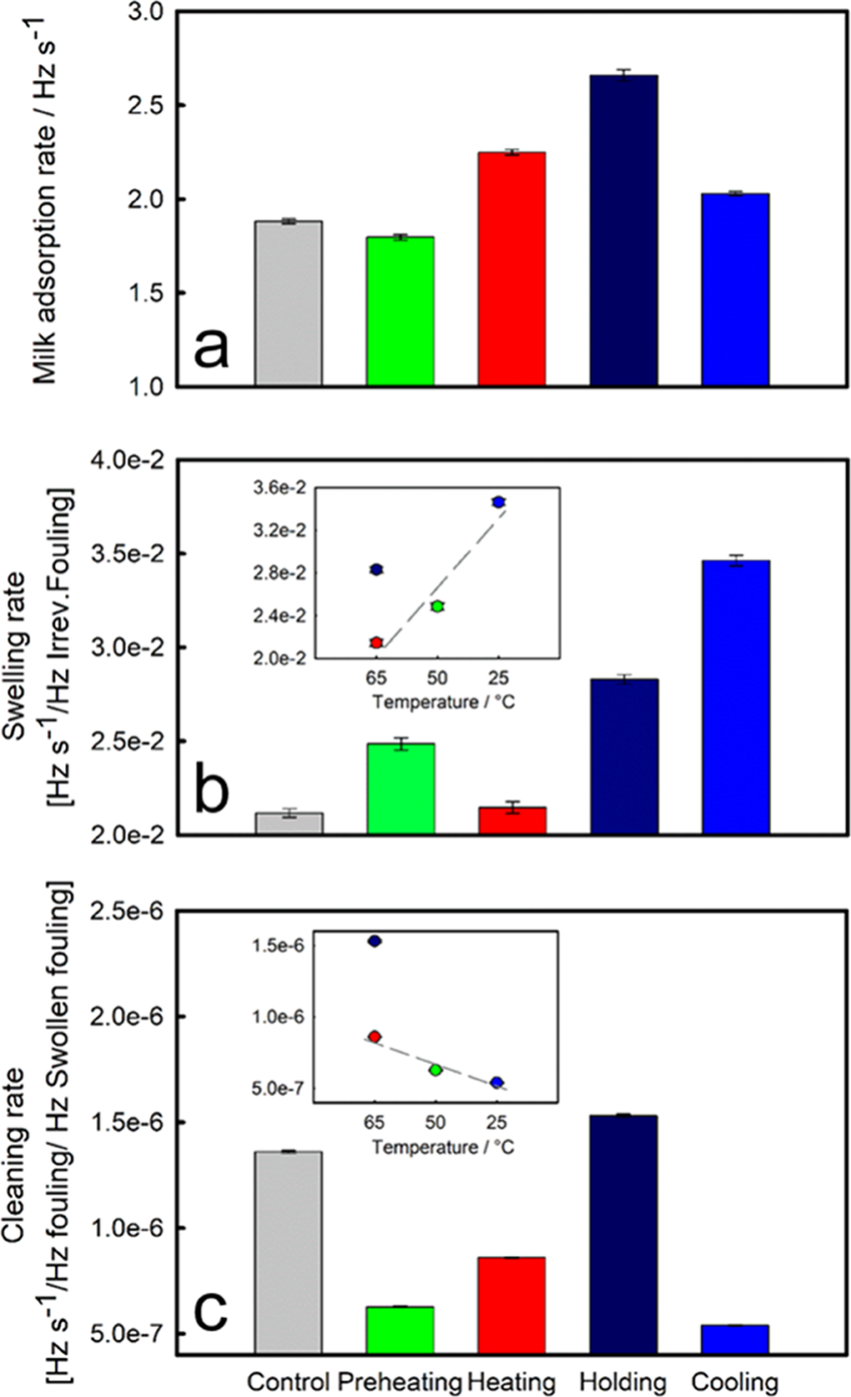
(a) Raw skim milk adsorption, (b) foulant swelling, and (c) cleaning rates as a function of the pasteurization stage. Rates (Hz s^–1^) were extracted from the slope (Δ*f* vs time) as detailed in Section 2.2.1 and the SI and normalized as a function of the Δ*f* value prior to the corresponding stage. Inset graphs show (b) foulant swelling and (c) cleaning rates as a function of surface temperature. During cleaning, the surface temperature was kept constant according to the one used for fouling formation. Error bars correspond to the standard error of at least two measurements.

Milk-surface interactions, as evidenced by the adsorption profiles, show a significant dependence on the profile of temperature applied. Figure 2a illustrates that increasing surface temperature (*T*_s_) from 50 to 65°C enhances the adsorption rate of milk from 1.80 ± 0.02 Hz s^–1^ (preheating) to 2.25 ± 0.01 Hz s^–1^ (heating). The adsorption rate was further increased to 2.66 ± 0.03 Hz s^–1^ (holding) when the temperature of the liquid (*T*_L_) was increased from 50 to 75°C, while *T*_S_ remained constant (65°C). It is worth noting that the adsorption rate was 2.03 ± 0.01 Hz s^–1^ when the liquid of 75°C was exposed to a surface of a low temperature (25°C). The changes in the adsorption rate clearly suggest that protein adsorption is dependent on both liquid and surface temperatures.

At saturation conditions (Δ*f* ≈ constant), the total adsorbed mass (Table 2) was also found influenced by the temperature profile: when *T*_L_ was kept under the denaturation temperature of β-Lg (≤65°C), adsorbed mass increased ca. 2.1 mg m^–2^ as *T*_S_ increased from 25 to 65°C (from control to heating). However, an increased *T*_L_ reduced the final amount of foulant or areal Sauerbrey mass adsorbed onto the metal surface (15.5 ± 0.8 and 15.1 ± 1.3 mg m^–2^ for holding and cooling, respectively), especially at low surface temperature.

**Table 2:** Combination of Liquid and Solid Temperatures (*T*_L_/*T*_S_) Used, Averaged Values of Frequency Shifts for Milk Adsorption (Δ*f*_adsorption_), Adsorbed Foulant Mass, Viscoelastic Ratio of the Adsorbed Film (Δ*D*_adsorption_/Δ*f*_adsorption_), Frequency Shift after Water Rinse (Δ*f*_water rinse_), Removal Percentage and Ratio, Irreversible Attached Foulant Mass, Swelling Frequency (Δ*f*_swelling_), Dissipation Shifts (Δ*D*_swelling)_, Solvation (Δ*f*_swelling_/Δ*f*_water rinse_), and Viscoelastic Ratio (Δ*D*_swelling_/Δ*f*_water rinse_) of the Irreversible Fouling Layer, Based on Overtones *n* = 7, 9, and 11^a^

condition	control	preheating	heating	holding	cooling
*T*_L_/*T*_S_ (°C)	25/25	25/50	50/65	75/65	75/25
Δ*f*_Adsorption_ (Hz)	90.7 ± 0.3	95.6 ± 5.8	102.6 ± 4.5	87.3 ± 4.7	85.4 ± 7.4
adsorbed mass (mg·m^–2^)	16.1 ± 0.1	16.9 ± 1.0	18.2 ± 0.8	15.5 ± 0.8	15.1 ± 1.3
Δ*D*_adsorption_/Δ*f*_adsorption_	0.19 ± 0.00	0.16 ± 0.03	0.12 ± 0.02	0.16 ± 0.03	0.26 ± 0.04
Δ*f*_water rinse_ (Hz)	44.9 ± 0.2	54.1 ± 0.6	54.0 ± 10.6	53.3 ± 0.7	36.3 ± 0.5
removal (%)	50.4	43.4	47.3	38.9	57.5
reversible removal ratio	145.9	164.3	126.1	73.0	132.2
deposit mass (mg·m^–2^)	8.0 ± 0.0	9.6 ± 0.1	9.6 ± 1.9	9.4 ± 0.1	6.4 ± 0.1
Δ*f*_swelling_ (Hz)	83.4 ± 0.1	96.8 ± 8.5	97.2 ± 11.4	101.3 ± 6.5	79.1 ± 7.6
Δ*D*_swelling_	10.7 ± 0.2	15.0 ± 1.1	10.0 ± 0.2	14.4 ± 1.7	13.8 ± 1.0
Δ*f*_swelling_/Δ*f*_water rinse_	1.86 ± 0.00	1.79 ± 0.14	1.80 ± 0.19	1.90 ± 0.10	2.18 ± 0.18
Δ*D*_swelling_/Δ*f*_water rinse_	0.24 ± 0.01	0.16 ± 0.01	0.10 ± 0.05	0.14 ± 0.01	0.18± 0.01

a Two repeats were at least carried out per pasteurization stage.

#### 3.1.2 Water Rinse

Surface adsorption of proteins involves both reversible and irreversible mechanisms.^32^ Following the fouling period (the first 15 min), a water rinse was performed for 10 min to remove any reversibly attached milk deposits. Figure 1 shows two characteristics once water was introduced:

(a) a continuously increased frequency, with a corresponding decrease in dissipation, suggests a steady removal process of surface foulant, as observed for the holding and cooling conditions, and
(b) some step-wise removal, shown by several distinctive stages in the recorded frequency/dissipation under preheating and heating conditions.

According to the frequency data (Figure 1a) during the water rinse period (15–25 min), the efficiency of removing the physisorbed foulant is 43.4, 47.3, 38.9, and 57.5% for preheating, heating, holding, and cooling conditions respectively (Table 2). The greatest removal ratios (Section 2.2.1) were obtained for the two least fouled conditions, preheating and cooling (164.3 and 132.2, respectively; Table 2), while holding condition, with the highest *T*_L_ and *T*_S_, showed the lowest ratio (73.0). It is assumed that the effect of *T*_S_ on the removal of the reversible fouling layer was negligible as rinse water effectiveness was not notably enhanced when temperature increases from 45 to 67°C.^33^

Following the rinse by water, the remaining surface foulant can be viewed as chemisorbed, or “irreversible fouling.” Deposit mass was quantified using the Sauerbrey equation (Table 2). When *T*_L_ was kept below the β-Lg denaturation temperature (≤65°C), the amount of chemisorbed foulant was similar after 15 min of processing for preheating and heating (9.6 ± 0.1 and 9.6 ± 1.9 mg m^–2^ respectively), 1.6 mg m^–2^ greater than that when *T*_S_ was kept at 25°C (control). However, as *T*_L_ increased (i.e., the holding and cooling experiments), the amount of surface foulant decreased; an increased *T*_S_ favored the final adsorbed mass (6.4 ± 0.1 and 9.5 ± 0.2 mg m^–2^ for cooling and holding, respectively).

#### 3.1.3 CIP Caustic Cleaning

Alkaline solutions are commonly used by the food industry to remove any proteinaceous deposits. Here, a chlorinated-caustic solution was introduced to the QCM-D chamber for removing the irreversible attached milk foulant. The surface temperature remained constant (Table 1) to avoid frequency and dissipation change due to temperature, and thus, viscosity changes.

Upon exposure to the cleaning solution, milk foulant swelled immediately to form a viscoelastic film, evidenced by the increased dissipation for all conditions studied (Figure 1b), followed by a gradually decreasing dissipation, alongside an increased frequency, both of which suggest a continuous removal of the deposit. Swelling rate (Section 2.2.1) shows a semilinear correlation with the surface temperature used in preheating, heating, and cooling conditions (Figure 2b), indicating that the low surface temperature (*T*_S_) could enhance swelling of the deposit.^34^ The decay region of the caustic removal (27 min onwards in Figure 1a) was modeled as a first-order process (Section 2.2.1) to establish the desorption kinetic that underpins the macroscopic cleaning efficiency.^35,36^ As with the correlation identified for the swelling phase, the rate of removal shows a semilinear trend as a function of the surface temperature (Figure 2c), where fouling removal was enhanced as *T*_S_ increases. Results obtained under the control condition (*T*_S_ and *T*_L_ at 25°C) show a low swelling rate (Figure 2b) but the highest viscoelastic ratio (Δ*D*_swelling_/Δ*f*_water rinse_ 0.24 ± 0.01; Table 2) and fast removal, indicating a poor molecular compaction and low-surface-adhesion strength of the deposit formed. In both cases (swelling and cleaning), deposit generated under holding showed an increased complex behavior, which is highly influenced by interconnected formation mechanisms that could affect both the deposit characteristics and its subsequent removal.

### 3.2 Physical Characteristics of the Surface Deposit

#### 3.2.1 Fouling Stage

During the deposition study, a notable variation in Δ*D*/Δ*f* between overtones was observed (Figure S2), which suggests the formation of a viscoelastic surface-adsorbed layer.^37^ This layer contains mostly calcium phosphate and protein as binding materials, of which protein adsorbs first due to its high surface activity.^38^ With the assumptions made by the Sauerbrey model (Section 2.2.1), the calculated areal mass density values in Section 3.1.1 may be slightly underestimated because not all of the adsorbed mass contributes to Δ*f* in viscoelastic systems.^39^ Therefore, we suggest that the differences between foulants could be attributed to the molecular packing during the build-up, which is controlled by the temperature at the interface:

(a) When *T*_L_ is below the denaturation point of proteins, surface fouling not only involves milk components adsorbing and saturating the stainless steel surface but also rearrangement in their interfacial configuration (Figure 1), which is significantly controlled by surface temperature, *T*_S_.
(b) Once *T*_L_ is increased to 75°C (holding and cooling conditions), the diffusion coefficient of the protein molecules in the bulk solution increases—ca. 10% according to the Stokes–Einstein equation (*D* = *k*_B_*T*/6πη*r*)—favoring surface adsorption and reducing the time required to reach surface saturation, where an increased *T*_S_ favors the chemisorption of milk compounds (i.e., holding).

**Figure 3:**
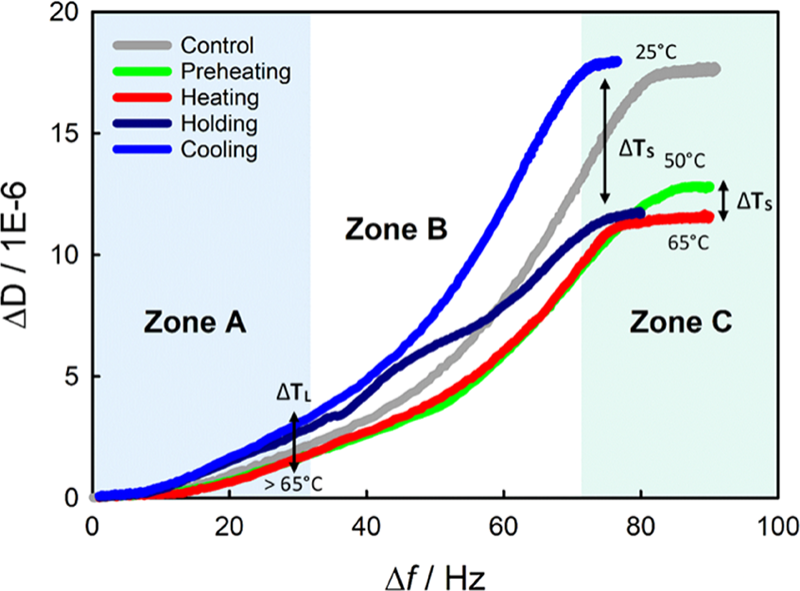
Dissipation vs frequency shift curves (Δ*D*/Δ*f*) where time is implicit. Lines show average data (two replicates) of milk adsorption as a function of the pasteurization section, using the overtones *n* = 7, 9, and 11. *T*_L_ and *T*_S_ indicate the temperature of the liquid (skim raw milk) and SS316 surface, respectively. Zones A, B, and C represent the initial adsorption of milk compounds, foulant conformational changes, and final configuration of the surface foulant, respectively.

To gain further insights into the characteristics of the adsorbed foulant, its viscoelastic properties were analyzed by examining Δ*D*/Δ*f* data (Figure 3) during milk adsorption, where time is implicit. The Δ*D*/Δ*f* curves show conformational changes over time owing to both liquid and surface temperatures:

- *Zone A*. Once the SS surface is exposed to the raw milk, surface adsorption is governed by the diffusion kinetics of individual milk compounds, initially independent of *T*_S_. Two adsorption characteristics were observed: (i) increased *T*_L_ from 25 to 50°C did not affect foulant properties; (ii) *T*_L_ above the denaturation point of proteins (75°C) enhanced the viscoelastic properties of the adsorbed foulant, suggesting adsorption of other softer bulk compounds such as aggregates of proteins, since unfolded β-Lg molecules could react with protein molecules or minerals to form aggregates in the bulk.^40^
- *Zone B*. Surface temperature begins to influence the fouling process. At low *T*_S_ (control and cooling), foulants present more viscoelastic properties. The formation process of low-temperature foulants differs from the high-temperature ones,^8^ leading to deposits with open structure and larger fat content.^8,41^ As *T*_S_ increases, deposits become more rigid, leading to the formation of a more compacted structure (low Δ*D*/Δ*f* values). Fouling under holding condition seems to follow a semilinear relationship between the adsorbed amount and its viscoelastic properties, suggesting that once the initially adsorbed layer of proteins is activated, there is a continuous mass transfer of compounds from the bulk fluid that favors the foulant build-up process.
- *Zone C*. The final foulant arrangement is clearly dependent on *T*_S_ that, in addition to favoring chemisorption of milk compounds, might favor the interfacial adhesion as well as the cohesion of the foulant over time. Film viscoelasticity, defined here as −Δ*D*_adsorption_/−Δ*f*_adsorption_ ratio (Section 2.2.1), was reduced when *T*_S_ increased from 50 to 65°C (0.16 ± 0.03 and 0.12 ± 0.02 respectively; Table 2), and slightly enhanced when the *T*_L_ increased (75°C; holding); reduced viscoelasticity suggests greater foulant compaction while increased viscoelastic ratio might indicate that there has been a significant mass transfer from bulk fluid compounds under holding condition as both stages are working at the same *T*_S_.

#### 3.2.2 Rinsing

**Figure 4:**
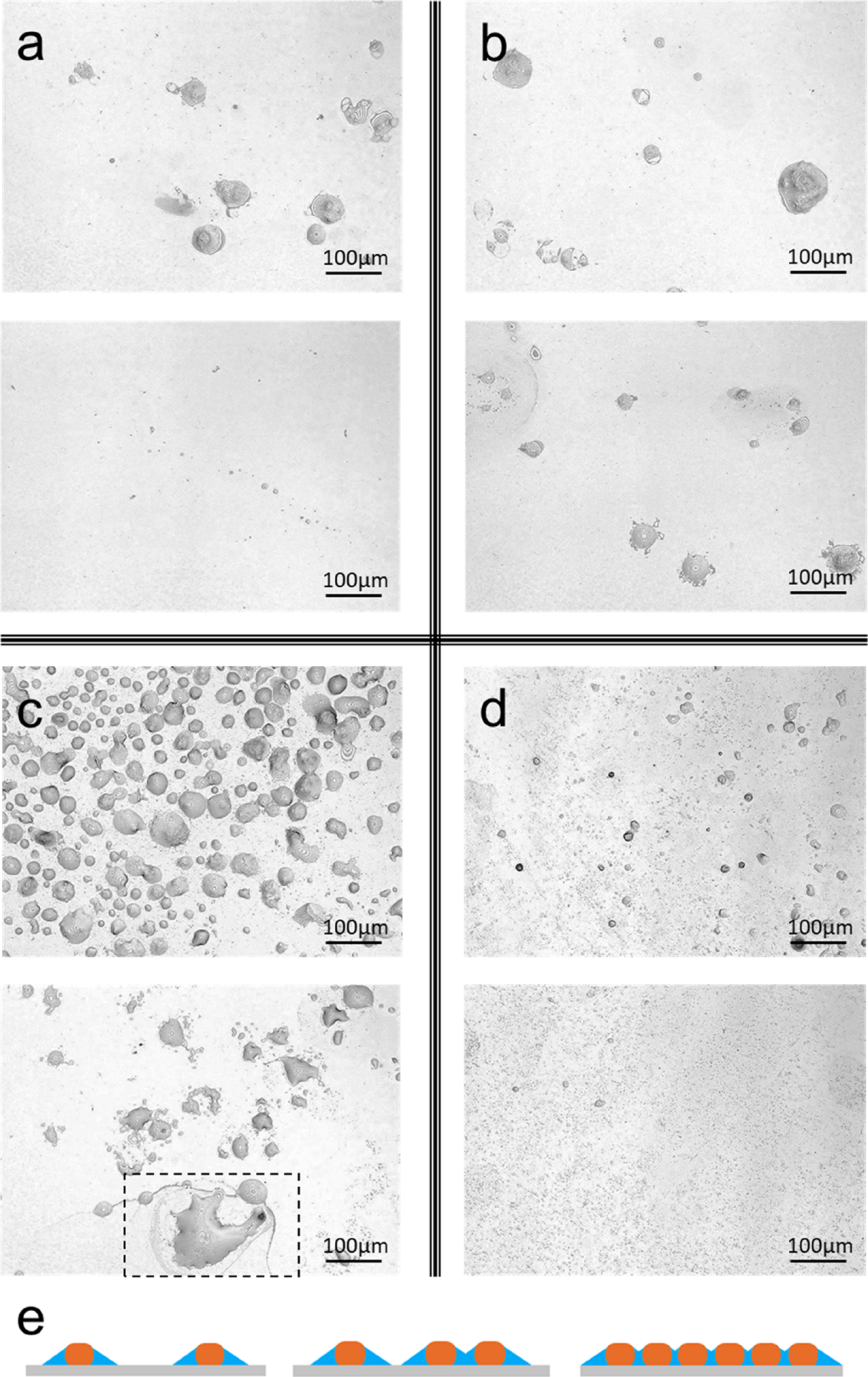
Surface morphology of milk-fouled QCM-D sensors, after being rinsed by water, for each pasteurization section: (a) preheating, (b) heating, (c) holding, and (d) cooling. Two surface images are showed per pasteurization section. Samples are characterized by 3D laser microscopy (magnification 20×). (e) Coverage dependence of solvent contribution to the QCM response; the fractional trapped liquid generally decreases with increasing coverage and can be rationalized as a coat (blue), which might overlap surrounding each deposit formed. The marked area of (c) shows a residual mark of the liquid coat that surrounded deposits amid pasteurization.

Following the water rinse stage, the surface morphology of the remaining chemisorbed foulant layer was acquired by 3D laser microscopy (Figure 4). Although all surfaces examined were covered with an irreversibly bound foulant (according to QCM-D data; Section 3.1.2), there is a distinction between the surface morphology of foulants generated under different conditions: a continuous particulate layer was produced under the cooling condition, isolated aggregates were formed under the preheating and heating conditions, while a combination of both of these characteristics were found under holding condition. Preheating and heating show deposits of similar structures. However, the number and size of the attached deposits were found to increase under the heating condition, implying that the fouled area was further developed. Some of those surface deposited were weakly attached to the SS, and removed by the water rinse, where Δ*D* (Figure 1b) dropped below zero due to the abrupt detachment of these deposits. When *T*_L_ (75°C) was above the denaturation point of β-Lg, surface temperature (*T*_S_) affected notably the structure and amount of the foulant deposited (Figure 4c), leading to an extended cluster-fouled area (mean diameter of 37.5 ± 24.7 μm). Magens et al. found that raw milk deposits, which appear generally uniform in composition (protein and mineral), can form blooming regions (<40 μm) in the fouling layer.^42^ Moreover, the higher interface temperature and longer residence time in the holding section can also increase the mean protein aggregate size (from 20 to 60 μm).^1^ When *T*_S_ was kept at 25°C, small particulate deposits cover uniformly the whole surface of the sensor (Figure 4d). It is therefore safe to conclude that protein denaturation and aggregation are enhanced in the near-wall region owing to high interfacial temperature, which could also be intensified by the laminar regime inside the QCM chamber (Reynolds <1;^14^) that might favor interactions at the thermal boundary layer.

Removal of physisorbed foulant supports our hypothesis that the interface temperature governs the molecular packing and subsequently the adhesive strength of surface foulant: low *T*_L_ and *T*_S_ would result in a stratified structure with the physisorbed molecules weakly bind to the stainless steel. Once *T*_S_ is high enough to activate surface reactions, the increased *T*_S_ facilitates a foulant layer with an improved compaction (lower viscoelastic ratio) and interface adhesion (lower irreversible removal ratio). Alongside the activation of the adsorbed layer, if *T*_L_ is high enough to favor chemical reactions between bulk fluid compounds, there could be a diffusion of protein aggregates that accelerates the overall fouling rate.

Although holding and cooling conditions appeared to result the highest surface coverage (Figure 4), they show low-frequency shift during pasteurization (Figure 1), which suggests that some other factors could influence the QCM response observed:

(1) *Hydrodynamic effects and the motion of surface-adsorbed foulants*(26,43,44) under preheating and heating conditions, as they might favor the amount of hydrodynamically trapped liquid that surrounds each deposit (box of Figure 4c), which will impact Δ*f* measurements. The exact contribution of the trapped liquid to the frequency response varies with surface coverage, deposit height-to-width ratio, internal liquid content, as well as the lateral organization of surface-bound material^26^ (Figure 4e).
(2) The existence of an *underneath nanoscopic foulant layer* that is beyond the detection capability of the 3D laser microscope as QCM-D sensors are very reflective.
(3) The *formation of protein aggregates in the bulk fluid* might also limit the number of proteins interacting with the metal surface and reduce fouling.^3^

Hypotheses 2 and 3 are further verified in Section 3.3.

#### 3.2.3 Cleaning Stage

**Figure 5:**
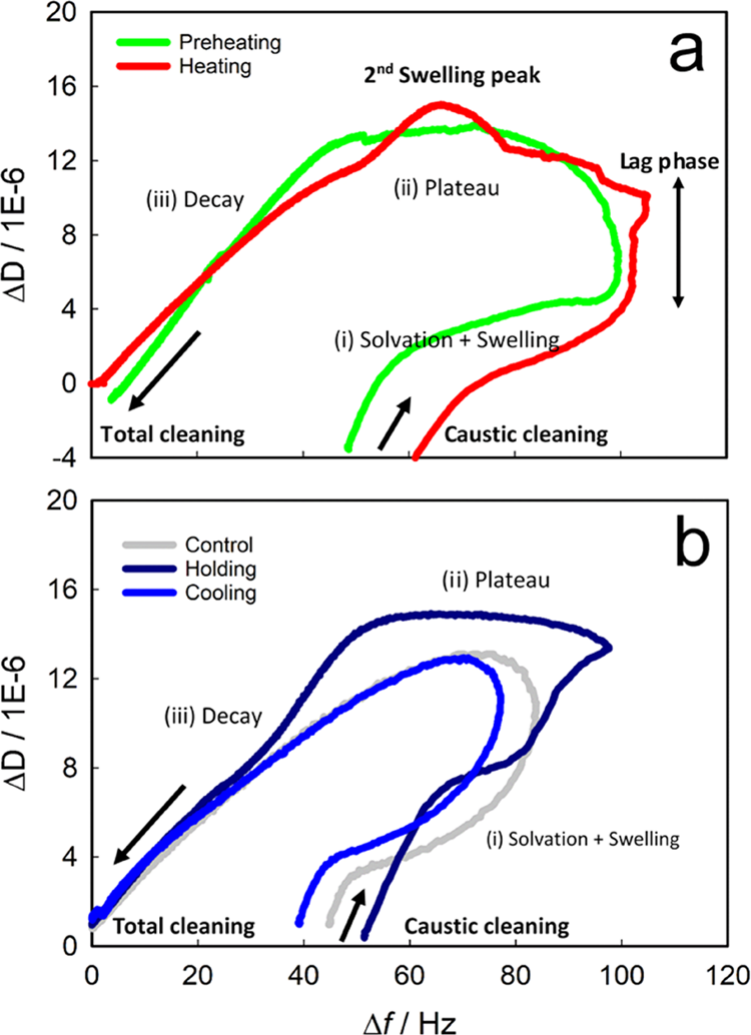
Dissipation vs frequency shift curves of average data (overtones *n* = 7, 9, and 11) amid caustic cleaning as a function of the pasteurization section: (a) preheating and heating, and (b) holding, cooling, and control test. The stages of solvation and swelling, plateau, and decay are indicated.

To better understand the effect of caustic cleaning on foulant mechanical properties, especially under holding condition, Δ*D*/Δ*f* results were analyzed (Figure 5) with a special focus on the stages of solvation and swelling, plateau, and decay. QCM results acquired under preheating and heating show similar cleaning mechanisms (Figure 5a): solvation and swelling of the fouling islands begin simultaneously; however, the maximum solvation ratio was reached before swelling was completed, resulting in a lag phase between the two peaks. When the maximum swelling was reached, there was a plateau of similar characteristics in both foulants before removal occurs by shear or mass transport (decay phase). As a point of interest, during the plateau, the heating foulant showed a second swelling peak, suggesting the formation of a more compact layer closer to the interface that, as mentioned in previous sections, is likely related to the higher *T*_S_ that favored deposit compaction (Δ*D*_swelling_/Δ*f*_water rinse_; Table 2). In fact, the reaction of NaOH with aged foulant material might be slower.^45^

Foulants generated under control, holding, and cooling conditions (Figure 5b) show similar cleaning mechanisms but slightly different from the previous pasteurization conditions because there was no lag phase between swelling and solvation. The viscoelasticity ratio (Δ*D*_swelling_/Δ*f*_water rinse_; Table 2) was especially enhanced for the deposits formed at high *T*_L_, supporting previous studies where heat-denatured whey proteins enhance water solvation, and especially, when protein aggregates are formed.^46−49^ The plateau stage was negligible for control and cooling foulants where the decay stage started quickly, but appreciable at holding, where a uniform swelling of deposit layer leading to removal by diffusion/shear was observed before its removal by shear/mass transport. Therefore, swelling and cleaning mechanisms, especially below pH 13, are closely related to the foulant formation conditions.^36,50,51^

### 3.3 Characterization of Nanoscopic Foulant Layer

As mentioned in Section 3.2.2, the presence of a molecular foulant layer and protein aggregation in the bulk fluid might affect QCM-D response as well as control fouling induction. To evaluate the effects of surface history and protein aggregation, a whey protein-based solution was used to foul stainless steel coupons in a customized flow cell. This solution was chosen for several reasons: (i) whey proteins are the main drivers for milk fouling at nanoscopic levels;^15^ (ii) the structure of WPC fouling is similar to that found for real milk between pasteurization temperatures of 42 and 120°C;^52^ (iii) whey proteins account for more than 50% of the fouling deposits under 100°C;^3^ and (iv) to minimize chemical heterogeneity. The same temperature profiles (Table 1) were used to prepare the model foulant on the stainless steel coupons using a customized flow cell (Section 2.3). The maximum surface temperature was set at 75°C rather than 65°C to better mimic the pasteurization conditions.

#### 3.3.1 WPC Fouling Induction and Nanomechanical Removal

WPC fouling formation process and the adhesion strength of the inductive foulant film were analyzed as a function of the set temperature during pasteurization using atomic force microscopy. WPC-fouled samples were collected every 2.5 min for a total time of 15 min.

Surface fouling could be classified into two stages:

##### 3.3.1.1. Preheating and Cooling

**Figure 6:**
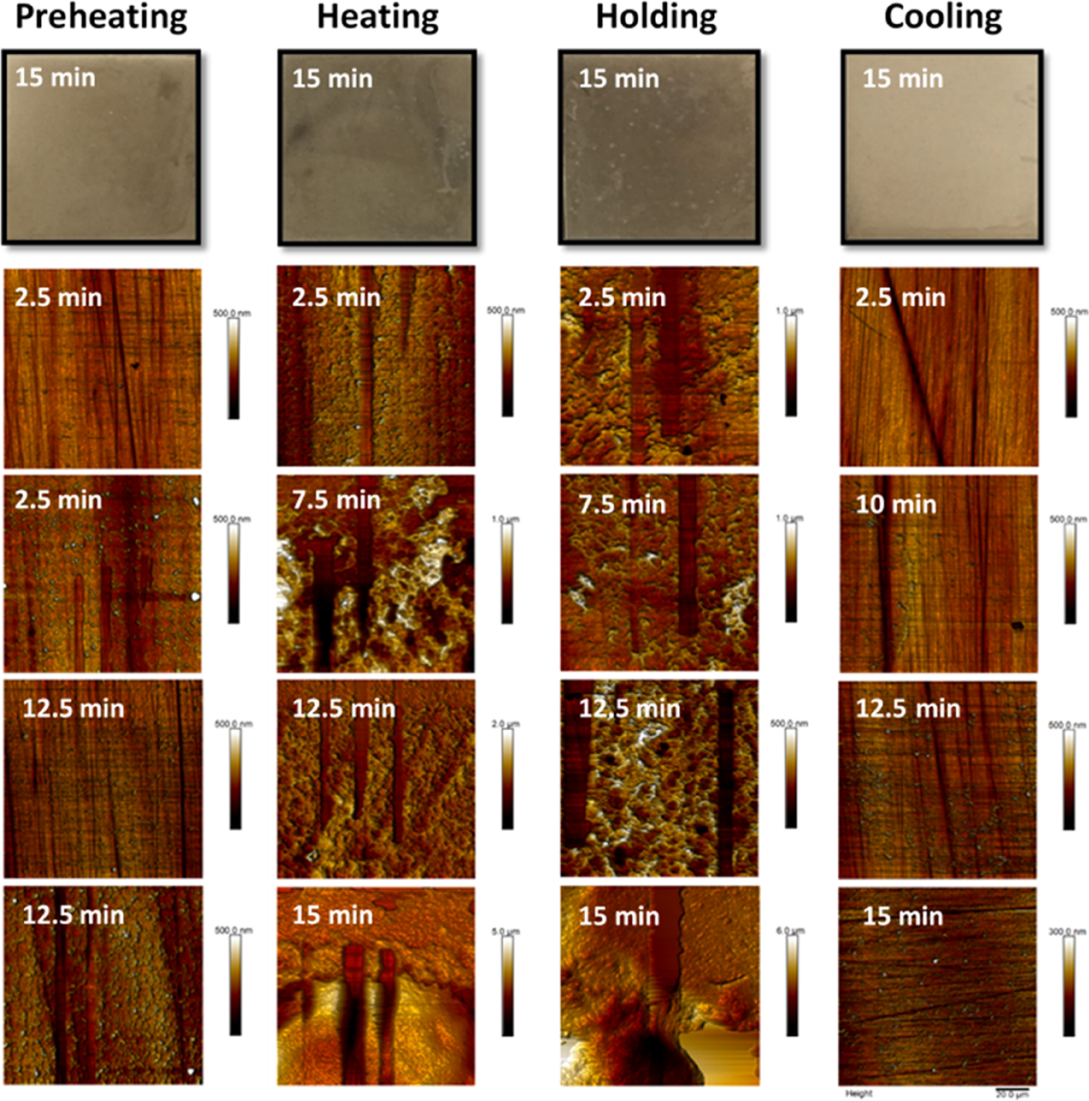
Surface morphology of WPC fouling, characterized by AFM, as a function of both exposure time and temperature profile: preheating, heating, holding, and cooling; temperatures are listed in Table 1. Top pictures show the WPC-fouled stainless steel surfaces (2.5 × 2.5 cm) after 15 min of pasteurization. AFM micrographs show an example of the growth sequence of milk fouling as a function of the pasteurization conditions. Straight scraping marks show the partial or total nanomechanical removal carried out using the AFM technique.

At preheating, two main areas can be easily identified: a homogeneous submicron film, and another with a significant deposition of foulant. The quasi-invisible foulant (Figure 6, **preheating2.5 min**) was formed by small clusters with an average height of 51.4 ± 36.6 nm and an overall surface roughness (*R*_a_) of 15.6 nm. These results agree with Jimenez et al.^15^ who observed unfolded protein clusters (not aggregates) of approximately 60 nm in diameter deposited homogeneously on the steel surface but formed at higher processing temperatures (62–92°C). The significantly fouled part shows different stages of fouling growth at short processing times (Figure 6, **preheating**): similar clusters to those mentioned above (58.1 ± 8.2 nm) within a uniform thin film (thickness of 30.9 ± 12.2 nm) that could be removed completely by an applied force of 31.2 μN. Other large particulate deposits scattered throughout the surface (e.g., 3.4 × 58.6 × 24.6 μm (H x W x L)), which may correspond to the isolated fouling observed in Section 3.2. As the size of the clusters increased (height increases from 82.9 ± 28.5 to 127.85 ± 52.8 nm), the structure became more compacted and smoother (*R*_a_ 31 nm), increasing the removal force to 43.6 μN. These results also agree with the surface layer (*R*_a_ 32 nm) composed of a juxtaposition of protein clusters of different sizes (40–100 nm) reported in ref (15). The film thickness barely increases over time (159.1 ± 93.6 nm at 15 min; Figure 6, **preheating 12.5 min**). These findings support previous claims that the initial phase of fouling begins with the formation of a homogenous proteinaceous layer on the stainless steel surface^9,15,53^ that, as in Section 3.1, most of the proteinaceous foulant is adsorbed in the first few minutes of processing.

Under cooling condition, fouling induction is negligible for 10 min (Figure 6, **cooling**). At longer times, a lumpy structure started to develop similar to that found for the first adsorbed foulant layer under preheating; Figure 6**(cooling 15 min)** shows the most fouled area identified. For most of the cooling samples, total removal was obtained using a scraping force of 31.2 μN. Under the preheating condition, there were randomly distributed particulate deposits (> 50 μm) after 15 min of processing. The reduced adsorption of cooling foulant might be related to either (i) the low *T*_S_ used (Section 3.1.1) and/or (ii) the high *T*_L_ that might favor aggregation of protein in the bulk fluid—which will be studied in Section 3.3.3.

##### 3.3.2.2. Heating and Holding

A surface temperature higher than the denaturation point of β-Lg affects the amount of fouling developed. At 2.5 min of heating (Figure 6), there is a thin film of thickness, 30.5–105.4 nm, that can completely be removed by 12.5 μN of applied force, which is much smaller than those needed to remove the deposits formed under preheating or cooling, indicating a possible reversible adsorption of foulant that may be detached by flow shear. However, thicker fouled areas (>582 nm) were also found, requiring removal forces greater than those of the AFM force range (>62.3 μN). Scattered large deposits were identified after 2.5 min. While those large deposits are of a similar height over time, the nanofoulant layer grows (Figure 6, **heating from 2.5 to 15 min)** and a more packed film is formed, varying surface roughness according to the packaging grade of the foulant layer (e.g., at 7.5 min of processing, *R*_a_ is 24.7, 43.0, 61.8, and 112.0 nm for layer thicknesses of 71.6 ± 24.2, 111.2 ± 37.5, 303.6 ± 125.4, and 516.9 ± 28.8 nm, respectively). The adhesion strength of the foulant layer depends on pasteurization time: a layer of thickness ∼300 nm requires removal forces of 18.7, 31.2, and 62.3 μN after 7.5, 10, and 15 min, respectively.

Under holding condition, the number of samples with deposit thickness below 100 nm is low, and fouling develops rapidly beyond the measurement range of AFM; the resulting deposits are thicker than the ones produced under heating conditions with the same formation time, owing to the mass transfer of aggregates from the bulk fluid favored by higher *T*_L_. Figure S3 shows the thickness of the removed foulant sublayer as a function of the force applied: a similar induction mechanism that is controlled by the surface reaction was found for heating and holding conditions. The rough foulant layers presented in Figure 6 (**heating and holding**) appeared to be thick, rough, and nonhomogeneous due to calcium in the milk, which is consistent with the previous work.^15^ The calcium content of the WPC solution (6 mg mL^–1^)—higher than those observed in milks 0.08–0.17 mg mL^–1,^^54^—might affect the compaction of the deposits. Additional information related to the wettability of fouled surfaces can be found in the SI.

#### 3.3.2 Nanomechanical Properties of WPC Deposits

**Figure 7:**
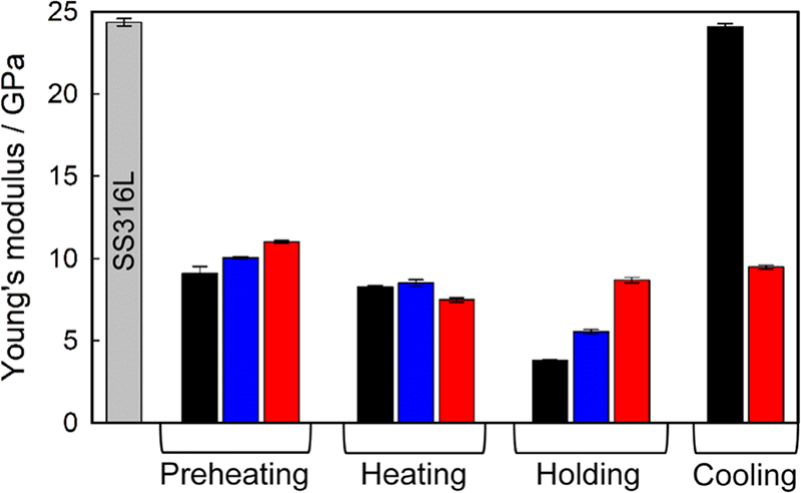
Young’s modulus of WPC fouling at 15 min of processing as a function of the pasteurization condition, from the least (black) to the most fouled area (red) defined by a microscopic inspection. Error bars show the standard error of at least 200 AFM force–distance curves.

Figure 7 shows the averaged values of the Young’s modulus of the milk foulants formed after 15 min under different conditions, from the least to the most fouled area. A reduced Young’s modulus was found from clean to postprocessing surfaces, confirming that the metal has been covered by the proteinaceous material. This layer becomes more rigid as fouling develops, likely influenced by the formation of more crosslinks^14^ that makes deposits more compact. As in Section 3.2, heating deposits are harder than preheating and holding, supporting previous observations that higher *T*_S_ enhanced deposit compaction, while *T*_L_ is yet relatively low. A high interface temperature (i.e., holding) could lead to the more flexible foulants—the lowest Young’s modulus of the four sections studied—as pointed out in Section 3.2. After 15 min of product cooling, most of the surface is still poorly covered, showing properties similar to that of the clean stainless steel; the most fouled cooling area corresponds to a random physisorbed particulate deposit. Additional information related to interfacial attraction and adhesion mechanisms can be found in the SI.

It is worth noting that the thickest foulant upon heating condition corresponds to the formation of an air bubble crater (Figure S4). This agrees with previous studies where the presence of air bubbles favors fouling.^35,42^ Although the bubble crater is the thickest deposit found, it is also the softest during heating (Figure 7, most fouled area), suggesting that there could be a faster but low-compacted growth mechanism highly influenced by the mass transfer from the bulk fluid.

#### 3.3.3 Formation of Bulk Aggregates

The increase of temperature of the bulk fluid might favor the formation of insoluble aggregates due to the heat sensitivity of minerals (e.g., calcium) and proteins,^8^ reducing fouling.^16^ To verify if there was aggregation in the bulk fluid at *T*_L_ 75°C that could reduce foulant adsorption (Section 3.2), processing time during cooling was prolonged up to 1 h. At the end of the experimental run, white and soft aggregate macrodeposits were found inside of the flow cell (see the SI), as a result of disulfide bonding.^55,56^ To quantify the cohesive bonding strength, deposits were extracted and characterized by micromanipulation (Section 2.6), showing a cohesive force of 22.3 ± 11.2 mN and a work per area of 1.8 ± 0.2 J/m^2^, within the range reported for swollen whey protein foulants.^57^ This reduced strength is likely related to the high water-holding capacity of the formed material^58^ that might also reduce foulant mass (reduced Δ*f* of holding and cooling conditions in Section 3.1). Therefore, at high *T*_L_, the formation of soft protein aggregates was favored, especially at long operational times, reducing foulant adsorption capacity (i.e., cooling (Section 3.3.1)) by limitation of the number of proteins interacting with the metal surface.

### 3.4 Comprehensive Mechanism of Milk Fouling Induction

**Figure 8:**
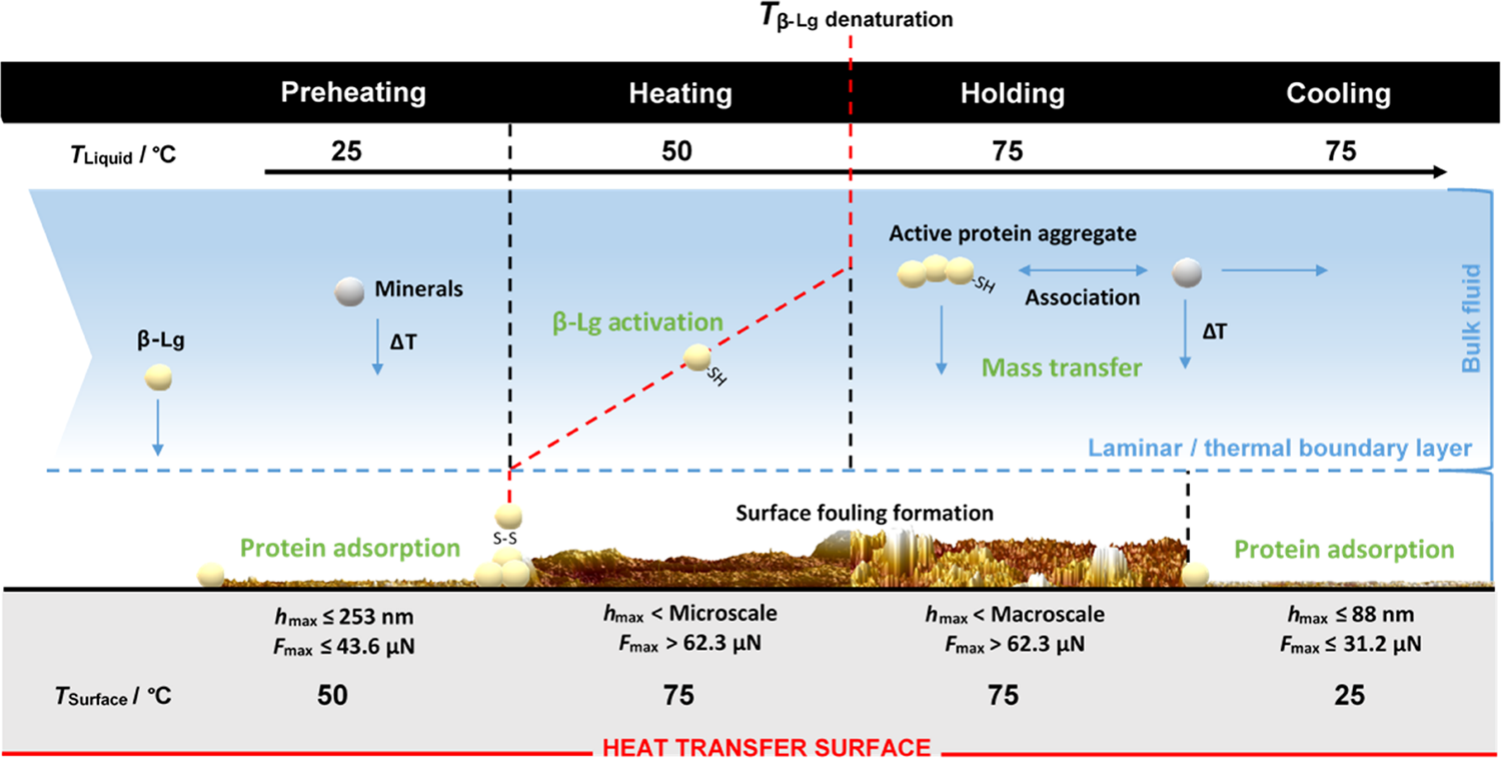
Schematic diagram of the proposed molecular mechanism of skim milk fouling induction (caseins are not included) as a function of the pasteurization temperature profile used under 75 °C for 15 min of processing. The pasteurization conditions studied are preheating, heating, holding, and cooling. Guide maximum values of removal force (*F*_max_) and thickness (*h*_max_) for the deposits formed are also indicated.

Building upon the fouling mechanism during milk thermal treatment,^59^ a detailed molecular mechanism (Figure 8) is proposed here using the comprehensive range of results obtained under controlled pasteurization conditions:

1 Milk fouling begins with an almost instantaneous adsorption of milk compounds, primarily small protein clusters,^9^ to cover the SS surface evenly (Section 3.1). This initial adsorption step is limited by the diffusion coefficient of individual milk compounds through the thermal boundary layer rather than the surface reaction itself, where low *T*_L_ and *T*_S_ (≤50°C) result in a stratified structure with physisorbed molecules weakly bind to the stainless steel (removed under 43.6 μN; Section 3.3). The proteinaceous layer is fully packed within the first minutes of pasteurization, and its thickness barely increases over time (Section 3.3.1), showing high water solvation capacity due to its poor compaction grade (Section 3.2), and it may also be more prone to subsequent protein binding than the bare SS substrate.^60^
2 During pasteurization, surface temperature governs the interactions in the near-wall area (thermal boundary layer), controlling the molecular packing during the deposit build-up (Section 3.2.1): *T*_S_ (≥ 65°C) above the denaturation temperature of β-Lg favors surface reaction (i.e., chemisorption of milk compounds), resulting in a compact foulant structure and increased adhesion to the SS surface over time (Section 3.3.1), which is reflected by the increased Young’s modulus (Section 3.3.2). The increased *T*_S_ would activate the adsorbed proteinaceous layer that favors mass transfer (e.g., proteins and minerals) from the bulk fluid, which is attributed to (i) limited quantity of unfolded proteins in the bulk fluid is sufficient to initiate fouling,^16^ (ii) the adsorbed foulant layer shows topographical similarities to that formed at higher processing temperatures,^15^ and (iii) the proteins of the first fouling layer have a secondary structure that differs from that of aggregates.^12^ Here, the presence of calcium could also influence the structural characteristics of the foulant as a function of both interface temperature and processing time since minerals diffuse through the proteinaceous foulant,^9^ enhancing cohesion between foulant layers.^7,10,11^
3 When *T*_L_ is above the denaturation point of β-Lg (> 65°C), the activated proteins in the bulk will react with each other and other species (i.e., minerals) to form large insoluble aggregates. These aggregates diffuse to the fouled solid surface, still activated due to the high *T*_S_, boosting the overall fouling rate (i.e., holding) and enhancing the viscoelastic properties of the deposits formed (Sections 3.2 and 3.3.2). However, if those aggregates are formed where *T*_S_ is low (i.e., cooling), there is a reduced surface adsorption capacity (Sections 3.1 and 3.3.1) that, along with little to no activation of the surface-adsorbed proteins, limits the number of compounds interacting with the metal surface, reducing fouling.^16,61^ Therefore, milk fouling phenomenon is rate-limited by either bulk reactions, mass transfer, or surface reactions depending on the temperature profile used for the treatment of pasteurization.

## 4 Conclusions

This work presented a molecular understanding of milk fouling process under various temperature profiles, which underpins different stages of a pasteurization process (preheating, heating, holding, and cooling). Our findings demonstrated that milk fouling kinetics, foulant characteristics, as well as the subsequent removal mechanism, are highly dependent on the temperatures used:

- *Milk fouling kinetics* is rate-limited by either bulk reactions, mass transfer, or surface reactions depending on the temperatures used: for low *T*_S_ (≤50°C) conditions (i.e., preheating and cooling), fouling begins with the adsorption of a proteinaceous layer, that upon its activation at *T*_S_ above denaturation point of β-Lg (i.e., heating), fouling develops by the mass transfer of milk compounds from the bulk fluid. However, high *T*_L_ (> 65°C, i.e., holding) favors aggregation in the bulk and aggregates diffuse to the previously fouled surface, which accelerates the overall fouling rate.
- *Mechanical properties of the foulant*: the foulant becomes more rigid as it develops due to an internal strengthening due to the formation of more crosslinks and, thus, a compacted structure. The deposit formed by surface reactions is harder because higher *T*_S_ enhanced deposit compaction, while *T*_L_ is relatively low. The deposit formed at a higher interface temperature (i.e., holding) is more flexible due to the adsorption of bulk aggregates onto the previously fouled surface.
- *Removal mechanisms*: the magnitude of adhesion force between foulant and substrate was enhanced with an increasing interfacial temperature and processing time. Furthermore, the force required to remove surface foulant would increase as a function of deposit thickness. During CIP, swelling and cleaning mechanisms are closely related to the foulant formation conditions, showing a semilinear relationship with surface temperature; higher *T*_S_ reduces swelling and enhances removal. The plateau stage is negligible for control and cooling foulants, where the decay stage starts quickly, but appreciable at holding, where a uniform swelling of deposit layer leading to removal by diffusion/shear is observed before its removal by shear/mass transport. On the other hand, for preheating and heating foulants, solvation and swelling begin simultaneously, reaching the maximum solvation ratio before swelling is completed, which results in a lag phase between the two peaks. When the maximum swelling is reached, there is a plateau before removal induced by shear or mass transport.

## Supporting Information Available

Detailed description of data analysis and experimental setup, scattering between QCM-D overtones during fouling formation, the relationship between deposit thickness and force required to remove heating and holding foulants using the AFM-based scratching method, surface adhesion measurements, analysis of protein aggregates formed in the bulk fluid, and study of the system wettability alteration throughout the induction period (PDF)
