# Supplementary material for: Molecular Understanding of Fouling Induction and Removal: Effect of the Interface Temperature on Milk Deposits
Source: ACS Appl Mater Interfaces. 2021 Jul 26;13(30):35506–17. doi: 10.1021/acsami.1c09553 (PMC8397245; doi:10.1021/acsami.1c09553)
Supplement: Supplementary file 1 — am1c09553_si_001.pdf [file am1c09553_si_001.pdf]

Supporting Information

Molecular Understanding of Fouling Induction and Removal: Effect of the Interface Temperature on Milk Deposits

Authors

Alejandro Avila-Sierra,[1,2] Holly A. Huellemeier,[2] Zhenyu J. Zhang,[1] Dennis R. Heldman,[2,3*] and Peter J. Fryer[1*]

Addresses

[1]School of Chemical Engineering, University of Birmingham, Birmingham B15 2TT, United Kingdom

[2]Department of Food, Agricultural, and Biological Engineering, The Ohio State University, Columbus 43210 Ohio, The United States of America

[3]Department of Food Science and Technology, The Ohio State University, Columbus 43210 Ohio, The United States of America.

Corresponding authors: Peter J. Fryer (p.j.fryer@bham.ac.uk) and Dennis R. Heldman (heldman.20@osu.edu).

1. QCM-D data analysis

Adsorption/desorption rates were extracted from three distinct phase: fouling, caustic swelling, and caustic decay. All rates (n = 2) were analysed using a default QR decomposition based linear least squares algorithm of MATLAB (MathWorks, Natick, MA, USA). Significant differences between rates were determined by non-overlapping 95% confidence intervals. To locate appropriate fitting regions, the following procedures were undertaken:

(1) Fouling rate: the linear fouling region was located by detecting local changes in slope between consecutive data points using the 'findchangepts' function in the MATLAB Signal Processing Toolbox.

(2) The effective area for the reversible CIP hydrodynamic removal was calculated by a default trapezoidal numerical integration method in MATLAB (MathWorks, Massachusetts, USA) with the "trapz" function. The selected times of integration were the start (first frequency data point with consecutively increasing frequency for a total of 900 seconds) and end of the water rinse.

(3) Swelling rate: due to the abrupt changes in frequency upon the introduction of caustic (**Figure 2**), the time point for the end of water rinse/start of caustic was manually selected. The swelling peak was identified using the 'findpeaks' function in the MATLAB Signal Processing Toolbox which identifies peaks based on differences in neighbouring data points.

(4) Decay rate: the decay region of caustic removal (**Figure 2**) was modelled as a first order reaction from the swelling peak to all following data points with a frequency less than 0 ($f < 0$): $f = f_0 e^{-kt}$, where f is frequency in (Hz), t is time (s), f_0 is a constant (Hz), and k is the decay rate (s^{-1}). This equation was linearized to enable the use of linear least squares fitting $\ln(f) = \ln(f_0) - kt$.

2. Microscopic flow cell

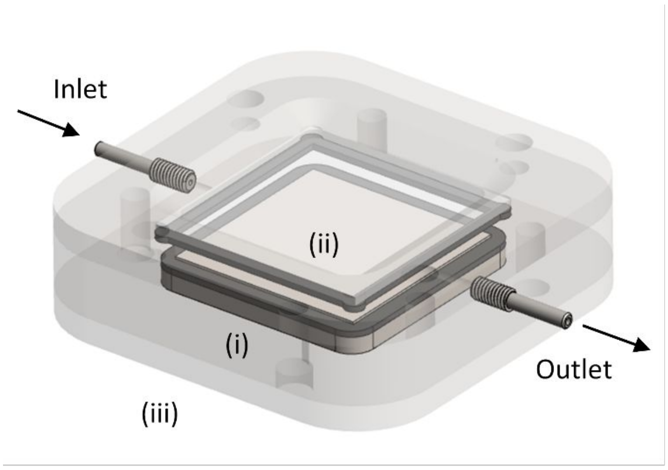

Figure S1. Schematic diagram of the flow cell used to simulate the solid-liquid interface of an industrial heat exchanger: (i) stainless steel surface, (ii) coverslip, and (iii) heating stage. The inner diameters of inlet and outlet tubes are 0.90 and 0.60 mm respectively.

The microscopic fouling setup is consisted of a flow cell with an integrated heating stage (temperature-controlled by a recirculating water bath) and a peristaltic pump that supplies a flow rate of 6.5 ml min^{-1}. The measurement chamber of the flow cell is 2.5 by 2.5 cm, and 3 mm high. The 316L stainless steel coupons (2.54 x 2.54 cm abrasively polished up to mirror finish Ra 0.03 ± 0.01 µm) were placed in the bottom part of the test cell. The top wall is made from glass, enabling visual inspection during the deposition process.

3. Contact angle measurements

Sessile drop method was selected to measure contact angle of a droplet (10 µL) of deionised water (18.2 MΩ cm) on mirror polished SS316L surfaces, before and after fouling formation (every 2.5 minutes). Equilibrium contact angle (ECA) was measured using an Ossila goniometer (Ossila Ltd, Sheffield, UK) in ambient conditions, followed by image processing using ImageJ software (National Institutes of Health, Maryland, U.S.A.).

4. Variations in QCM overtones data

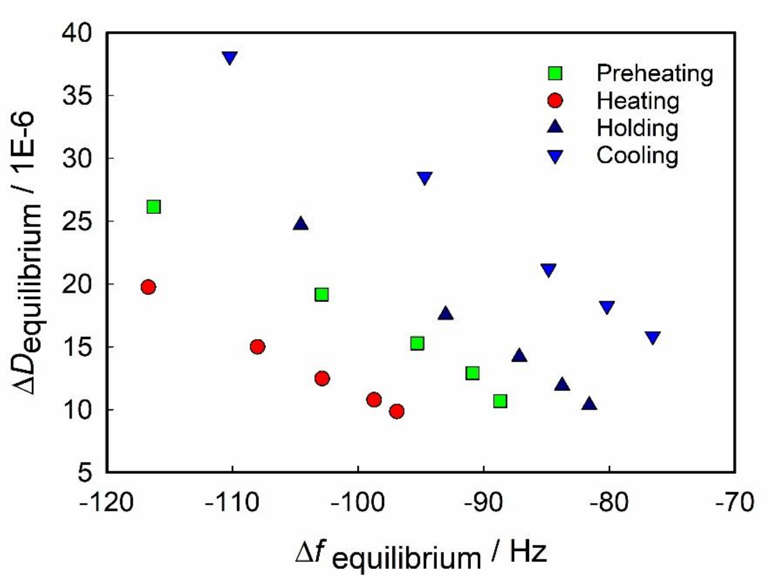

Figure S2. Scattering between overtones (n = 3, 5, 7, 9, and 11) at the state of equilibrium ($\Delta f \approx$ constant) upon milk adsorption.

5. Foulant thickness vs removal force

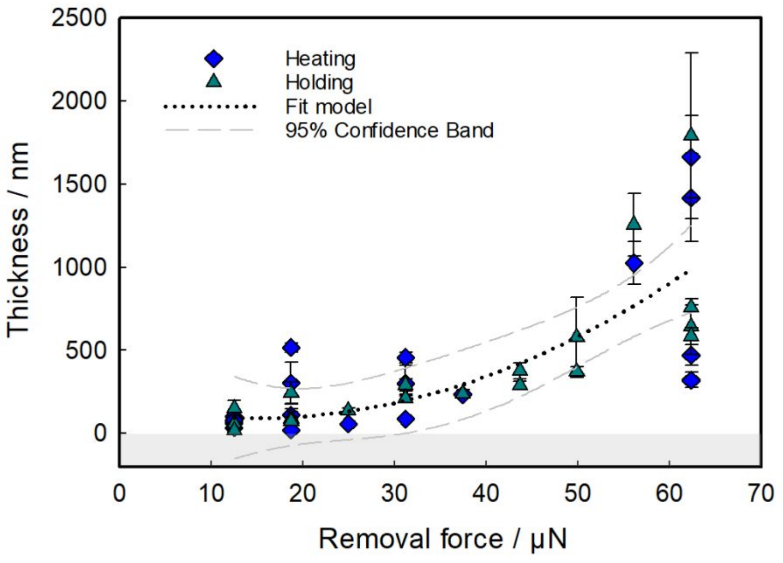

Figure S3. Relationship between deposit thickness and force required to remove, using the AFM based scratching method. Two conditions, Heating and Holding, are presented. Total time analysed was up to 15 minutes. Black dotted line show the fit model ($h\ (F) = y_0{+}a\ F{+}b\ F^2$) of holding deposits. Dashed grey lines show 95% confidence band.

A 2nd grade polynomial fit model has been applied to holding foulant to facilitate data visualisation:

$$h(F) = y_0 + a\,F + b\,F^2 \qquad R^2\ 0.7 \qquad \textbf{Eq. S1}$$

Where h is the thickness of the sample (nm) and F the maximum force applied (nN) to achieve the total removal of the deposit.

	**Coefficient**	**Std. Error**
y_0	183.8	283.6
a	-12.0	18.0
b	0.4	0.2

Table S1. Fitted parameters and standard deviation.

6. Surface adhesion measurements

The adsorbed protein clusters can be viewed as colloidal particles of which the principal factors determining adhesion to a solid substrate are (i) van der Waals forces, (ii) electrostatic forces, (iii) hydrogen bonding, (iv) hydrophobic effects, as well as other secondary factors such as (v) surface roughness, (vi) extension of the contact area through elastic or plastic deformation, and (vii) the presence of other materials (e.g. fats) [1]. Of the secondary factors, the effects of both surface roughness and the presence of other materials are negligible owing to the use of a sharp AFM tip (**section 2.5**) and a consistent whey protein based solution (**section 2.1**). Therefore, the purpose of this section is to analyse of interfacial interactions (i.e. attractive and adhesive forces) of the fouled surfaces at 15 minutes as a function of the pasteurisation condition. AFM force spectroscopy mode is used here (**section 2.5**): AFM cantilever tip is moved vertically towards the sample of interest (approaching stage) until to make contact between both bodies (contact phase), from which the tip is retracted (retraction stage) and moved to a different position. **Figure S4** shows a schematic representation and examples of force-distance curves acquired in air.

During the approaching phase, the main force gradient (i.e. van der Waals and electrostatic forces) is larger than the effective elastic constant of the cantilever tip, making the cantilever "jumps" onto the surface, in where two distinctive attraction mechanisms can be visualised (**Figure S4a-d**): rectangular- and V-shape attraction curves. In the case of hydrophilic surfaces such as SS316L (~ 100 nN at < 10 nm), a marked V-shape of moderate attraction at short range

occurs. A similar behaviour is also observed at Cooling surface (**Figure S4d**) that indicates its poor covering grade after 15 minutes of processing. These weak attraction forces are due to the meniscus force exerted by a thin layer of water vapour adsorbed on the sample surface, which barely affects attractive forces but prevents the tip from pulling off from the surface due to its high surface energy [2]. Other different attraction mechanism can be observed at Preheating, where attractive interactions show a more rectangular-shape of ~200 nN force that act at longer distance range (40-60 nm) from the surface. These long-range interactions are due to the adsorbed protein molecules onto the surface: at long distances (> 100 nm) the tip is far away from the surface and the deflection is zero. At 60 nm from the surface (similar distance that dimeter of protein cluster analysed in **section 3.3.1**), the tip contacts the fouled surface resulting in an initial attractive force. After this first jump, the force is fairly constant as the tip is penetrating through the poorly compacted foulant layer, followed by the contact line once the tip contacts a hard surface (up to 500 µN). Poorly fouled areas of different pasteurisation coditions (i.e. Holding) showed similar attraction mechanisms. As fouling develops (i.e. Heating and Holding), more marked V-shape interactions are visualised occurring at shorter distance range (< 30 nm), that might indicate again an increased surface covering hydrophilicity that allows the presence of capillary forces [3].

Figure S5 shows average adhesion forces of the surfaces tested, from the least to the most fouled areas. Averaged adhesion forces show slight differences within a reduced force range, in which for most of the cases a second adhesive jump (**Figure S4**) can be observed during retracting due to the stretching of foulant molecules that, given similar separation lengths for these samples, indicates the same type of stretched molecules, proteins. Upon contact of the processed liquid with SS, surface adhesion increased. At Preheating (**Figure S5a**), poor compaction of the adsorbed proteinaceous layer led to widely scattering forces (1.5 - 3.7 µN) that was significantly reduced as the adsorbed foulant become a more packed film; the major

number of adhesion events occurred at ~1.6 μN. At Heating (**Figure S5b**), adhesion is slightly reduced (1.5 μN) surely related to the increased foulant compaction (higher Young's modulus) mentioned in **section 3.3.2**, which reduces the contact area between the AFM tip and deposit. At higher interfacial temperature, i.e. Holding (**Figure S5c**), adhesion increased (~1.7 μN), as well as its distribution range (1.4 – 2.1 μN), surely influenced by the enhanced foulant viscoelasticity. Poorly covered areas such as cooling still showed similar adhesive properties to the pristine metal substrate.

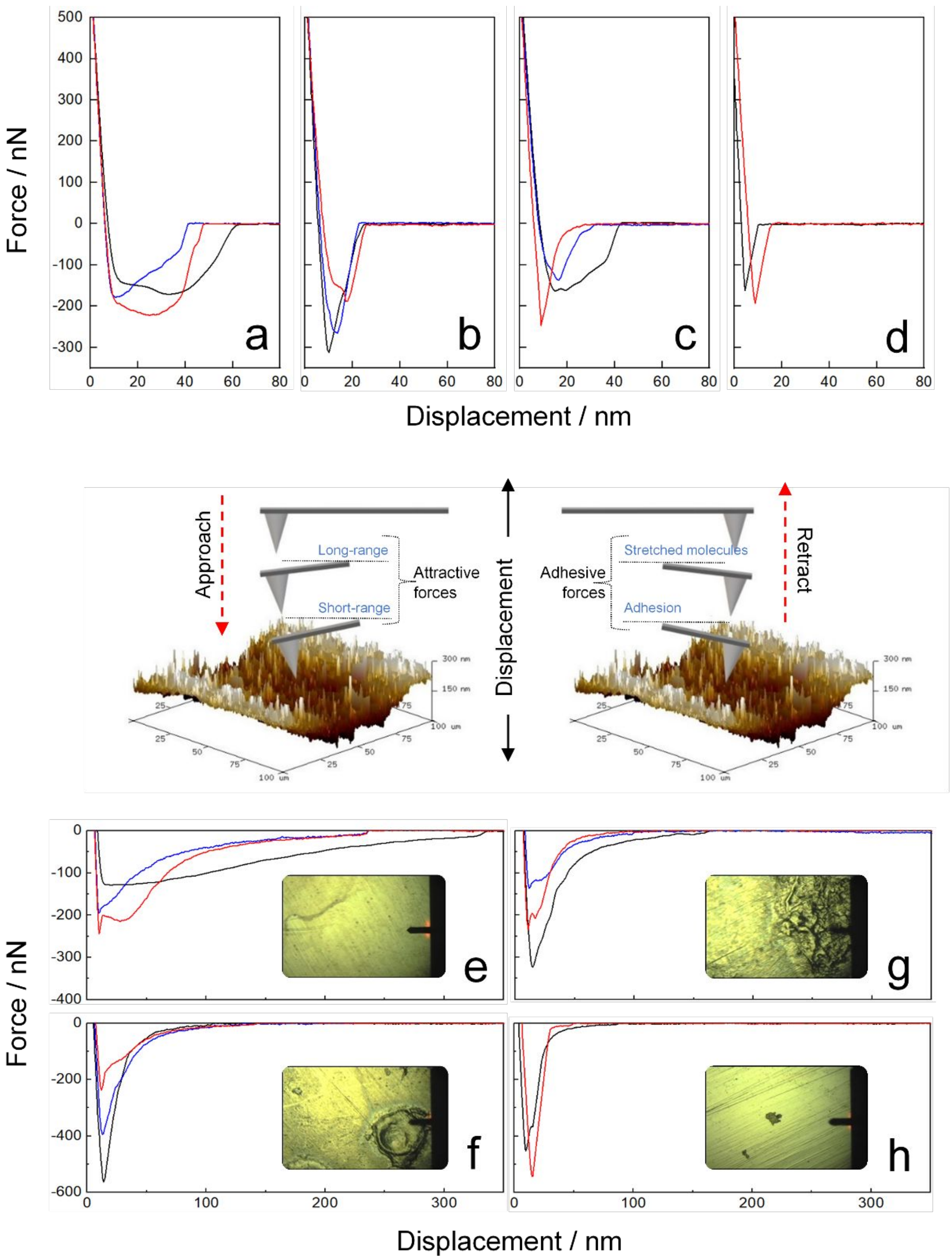

Figure S4. Representative force-distance interactions between the AFM tip and the deposits of interest formed after 15 minutes of pasteurisation. Approach (a, b, c and d) and retraction (e, f, g and h) curves as a function of pasteurisation section: Preheating (a, e); Heating (b, f); Holding (c, g) and Cooling (d, h). Lines show the forces involved from the least (black) to the most fouled area (red) of each sample. In the centre, there is a schematic diagram of the vertical tip movement during force-distance measurements.

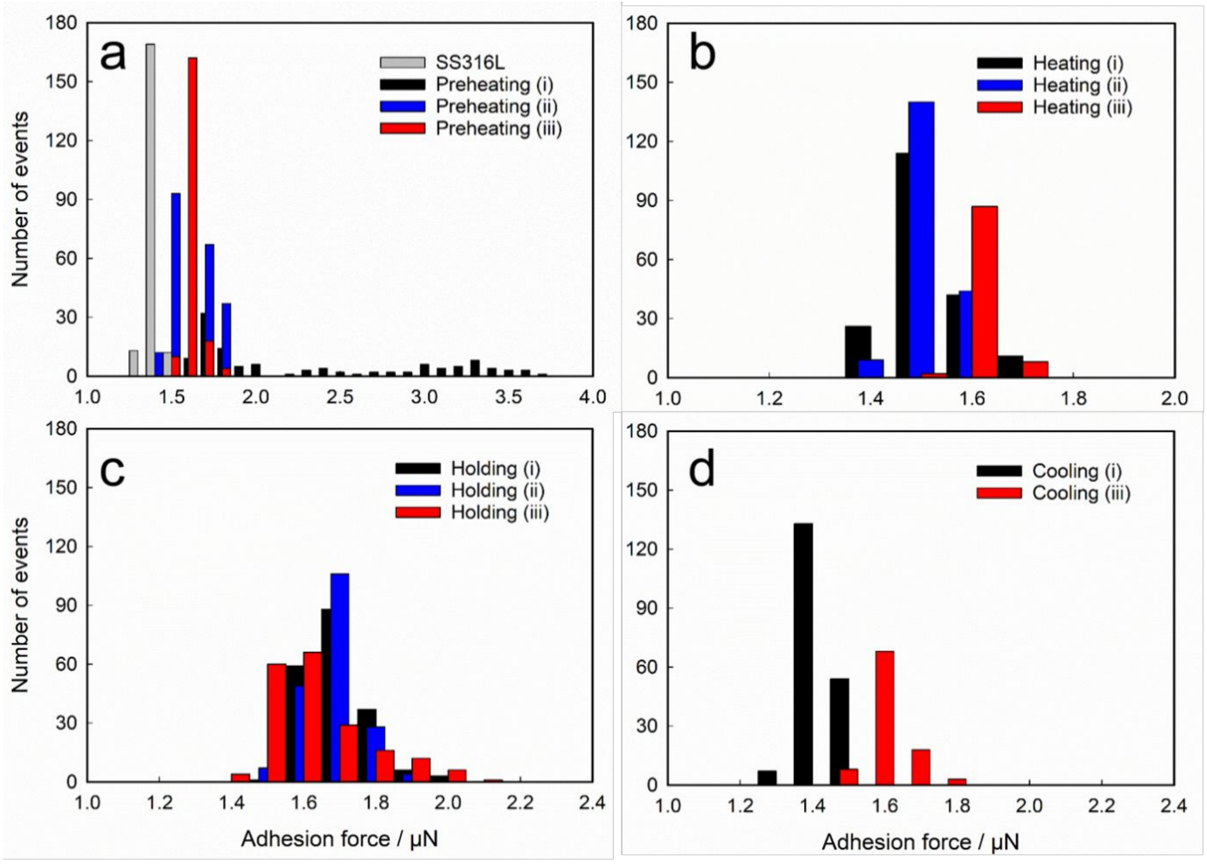

Figure S5. Adhesion force distributions between AFM tip and WPC foulant at 15 minutes of processing as a function of both the pasteurisation section (Preheating (a), Heating (b), Holding (c) and Cooling (d)) and fouling level, from the least (i) to the most fouled area (iii) defined by a microscopic inspection. Adhesion force of the clean stainless steel surface is also showed in graph (a).

7. Protein aggregation in bulk solution

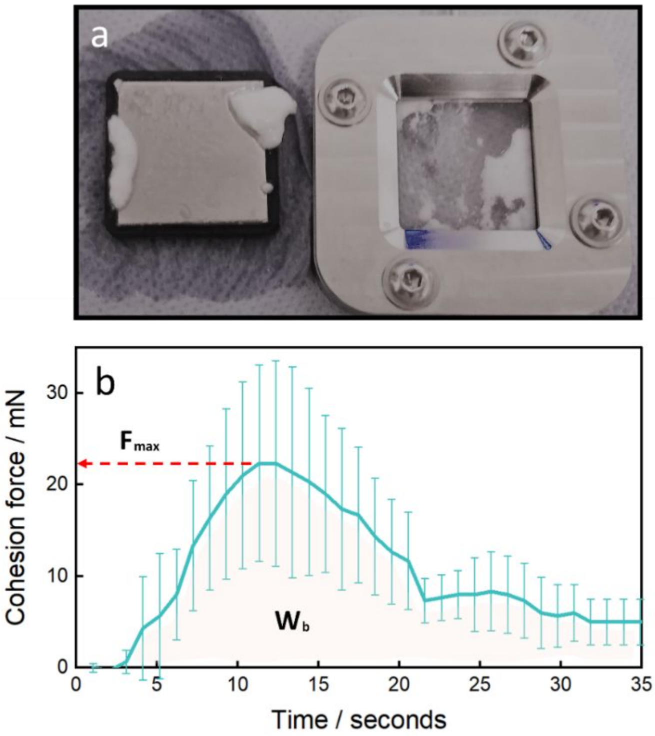

Figure S6. Formation of protein aggregates in the bulk fluid. Picture (a) shows an example of the deposits formed inside of the flow cell. Graph (b) shows the cohesive bonding of the deposit: cohesive force versus micromanipulation time. The parameters extracted are the maximum force applied (F_{max}) and the work done per unit of area (W_b).

8. System wettability alteration throughout the induction period

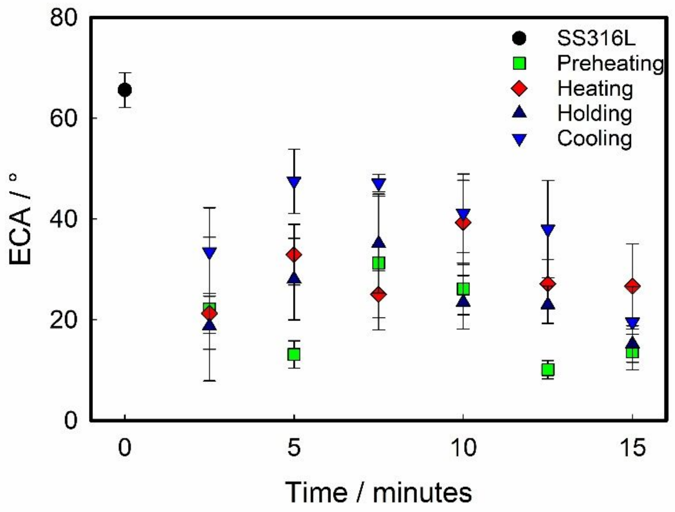

Figure S7. Equilibrium contact angle of water upon stainless steel surfaces, before and after foulant deposition, as a function of processing time. Samples are characterised every 2.5 minutes up to a maximum time of 15 minutes. The four pasteurisation stages studied are Preheating, Heating, Holding and Cooling. Error bars show the standard deviation of at least three different droplets.

During pasteurisation, alterations of the underlying surface chemistry due to adsorption of milk compounds might affect the interfacial adhesion between the processed liquid and the contact surface [4], where especially hydrophobic effects play a role through acid-base interactions [1]. To assess surface wettability from a macroscopic level, equilibrium contact angle (ECA) of water droplets was quantified (method detailed in **section 3** of **SI**) as a function of both pasteurisation section and processing time (up to 15 minutes). In all sections, ECA follows similar wetting behaviour: it decreases drastically after being surfaces exposed to the bulk fluid, increasing afterwards up to times of 5-10 minutes, to decrease again as fouling develops. The initial decrease might correspond to the initial contact between the whey protein complex and the stainless steel surface of **section 3.1**, in where foulant adsorption is uncompleted and the proteinaceous layer is still poorly packed, favouring water penetration and the subsequent distortion of the droplet contact line. Once it is packed (5-10 minutes), ECA increased. Then, ECA begins to decrease as surface foulant develops, surely relate to the enhanced hydrophilicity of the covering surface material (**section 6** of **SI**). At Cooling, ECA values were closer to the value obtained for the clean SS, supporting previous observations where it was the poorest fouled section. Overall, surface wettability is significantly altered as foulant is adsorbed and fouling develops, depending on both the pasteurisation conditions (i.e. temperature profile) and the processing time.

References

[1] Visser, J. Particle Adhesion and Removal: A Review. Part. Sci. Technol. 1995, 13, 169–196. https://doi.org/10.1080/02726359508906677.

[2] Cappella, B.; Dietler, G. Force-Distance Curves by Atomic Force Microscopy. Surf. Sci. Rep. 1999, 34, 1–104.

[3] Harrison, A.J.; Corti, D.S.; Beaudoin, S.P. Capillary Forces in Nanoparticle Adhesion : A Review of AFM Methods. Part. Sci. Technol. 2015, 33, 526–538. https://doi.org/10.1080/02726351.2015.1045641.

[4] Avila-sierra, A.; Zhang, Z.J.; Fryer, P.J. Effect of Surface Roughness and Temperature on Stainless Steel - Whey Protein Interfacial Interactions Under Pasteurisation Conditions. J. Food Eng. 2021, 301, 110542. https://doi.org/10.1016/j.jfoodeng.2021.110542
